# Relationship between Caregivers’ Smoking at Home and Urinary Levels of Cotinine in Children

**DOI:** 10.3390/ijerph111212499

**Published:** 2014-12-01

**Authors:** Yun Wang, Mei Yang, Lang Tian, Zhiqiang Huang, Faming Chen, Jingsong Hu, Fuzhi Wang, Gui Chen, Shuiyuan Xiao

**Affiliations:** 1 Department of Social Medicine and Health Management, School of Public Health, Central South University, Changsha, Hunan 410078, China; E-Mails: xjwangyun@csu.edu.cn (Y.W.); Ym8342@163.com (M.Y.); bengbu_wangfuzhi@126.com (F.W.); cgwkkk@163.com (G.C.); 2 School of Nursing, Xinjiang Medical University, Urumqi, Xinjiang 830000, China; 3 Department of Pediatrics, the Third Xiangya Hospital of Central South University, Changsha 410013, China; E-Mail: tianlangdoc@163.com; 4 Academy of Inspection and Quarantine, Changsha 410004, China; E-Mail: huangzqq@126.com; 5 Changsha Central for Disease Control and Prevention, Changsha 410001, China; E-Mails: cfm@cscd.gov.cn (F.C.); hjs009@126.com (J.H.)

**Keywords:** smoking, caregiver, environmental tobacco smoke (ETS), children, gas chromatography-triple quadrupole mass spectrometry (GC-MS/MS), cotinine

## Abstract

*Objective*: To assess the impact of different smoking behaviors of caregivers on environmental tobacco smoke (ETS) exposure in children aged 5–6 years in Changsha, China. *Methods*: We conducted a cross-sectional, random digit-dial telephone survey of caregivers (*n* = 543) between August and October 2013. Caregivers’ smoking behaviors were collected by a questionnaire. Exposure assessment was based upon determination of urinary cotinine levels in children employing gas chromatography–triple quadrupole mass spectrometry (GC-MS/MS). *Results*: In children not living with a smoker, children living with one smoker, and children living with more than one smoker at home, median urinary cotinine concentrations (ng/mL) were 0.72, 2.97, and 4.46, respectively. For children living with one smoker, median urinary cotinine levels of children exposed to ETS were associated with caregiver smoking behaviors, *i.e.*, if a caregiver consumed more cigarettes (>20 compared with ≤10; 7.73 *versus* 2.29 ng/mL, respectively). *Conclusions*: The magnitude of ETS exposure in children is correlated with the smoking behaviors of the caregiver. Counseling for smoking cessation and educational interventions are needed urgently for smoking caregivers to increase their awareness about ETS exposure and to encourage smoking cessation at home or to take precautions to protect children’s health.

## 1. Introduction

Environmental tobacco smoke (ETS) is one of the most common indoor air pollutants. There are two components attributed to ETS, second-hand smoke and third-hand smoke [1]. Second-hand smoke is complex mixture of chemicals generated during the burning and smoking of tobacco products [2], while third-hand smoke consists of residual tobacco smoke that adsorbs to indoor surfaces and remains after the majority of the airborne components of the smoke have cleared [3,4,5].

ETS is one of the most important and toxic exposures to children found in indoor environments. Globally, 40% of children are exposed regularly to second-hand smoke, and 28% of deaths from second-hand smoke occur in children [6]. Children exposed to ETS are at an increased risk of sudden infant death syndrome, acute respiratory infections, ear infections, and severe asthma [7,8,9,10,11]. The exposure rate of children may be significantly higher in China. For example, Yao *et al.* found in 2008 that at-home ETS exposure rates were 68% among children aged 0–18 years among six rural counties of China [12].

The government of the People’s Republic of China instigated a ban on smoking in any indoor public facility on 14th February, 2011 [13]. Nevertheless, these bans did not extend to protecting individuals exposed to ETS in their home environment [14]; an even greater concern exists for children, whose exposure is strongly associated with parental smoking [15]. Data from one national survey revealed that, of 540 million Chinese people who are exposed to ETS, 150 million are children aged <15 years [16]. Infants and very young children cannot complain; older children who are affected by ETS may not complain, or may be ignored or reprimanded if they do. Furthermore, children often cannot remove themselves from the exposure and are therefore dependent on other measures for protection [17].

The best way to protect children exposed to ETS is to eliminate smoking in indoor spaces, including homes and private vehicles. Comprehensive and evidence-based tobacco control programs can substantially reduce the rate of ETS exposure in children, especially parental smoking behavior interventions. For example, a randomized clinical trial has previously suggested that the intervention group had significantly lower air nicotine levels at home, with a 17% increase in the prevalence of caregiver-reported home smoking bans, and a 13% decrease in caregiver smokers compared with the control group [18].

The measurement of ETS exposure in children is not simple. ETS studies for children rely on self-reported measures given by their parents or caregivers, who are likely to be either the source of ETS or responsible for it [19]. However, this type of exposure assessment is indirect and imprecise. More precise measurements are obtained from active monitoring and the use of biomarkers (typically cotinine) in the saliva, urine, blood and hair samples [20]. Cotinine is accepted as the best available biomarker of exposure to ETS [21]. It is an alkaloid found in tobacco and a metabolite of nicotine, with a half-life of 20 h. Cotinine remains stable with temperature changes, and has high levels of specificity and sensitivity [22]. Urinary cotinine studies are frequently used because of the non-invasive methodology. Therefore, the measurement of cotinine levels in body fluids together with caregiver-completed questionnaires could be an optimal method for assessing ETS exposure in children. To the best of our knowledge, no studies have been performed that measured the impact of smoking behaviors by Chinese smokers at home on children exposure to ETS using urinary levels of cotinine as an objective parameter.

The aim of this study was to assess the relationship between caregivers’ smoking at home and urinary levels of cotinine in children aged 5–6 years old. We hypothesized that the urinary cotinine levels of children who were exposed to ETS would be significantly higher compared with those of children who were not exposed to ETS at home.

## 2. Materials and Methods

### 2.1. Ethical Approval of the Study Protocol

Written informed consent was obtained from the caregivers of the study subjects to be included in the study. All caregivers voluntarily joined this study and gave their written informed consent. The caregivers mainly included the father, mother and others, such as grandparents or siblings. Written informed consent was also obtained from the parents of the children involved. The kindergartens and teachers of the children were informed about the study by Changsha Center for Disease Control and Prevention and School of Public Health of Central South University. The study was conducted in accordance with the Declaration of Helsinki, and the protocol complied with the current laws of China. The study protocol was also approved by the Ethics Committee of the Clinical Pharmacology Research Institute of Central South University (Changsha, China; approval ref: CTXY-12023).

### 2.2. Study Population and Procedures

The present study had a cross-sectional design and data were collected between August and October 2013. The study cohort was selected using two-stage, simple random sampling. In the first stage, districts in Changsha were identified to include six districts and one county; three districts and one country were drawn randomly. In the second stage, preschools in Changsha were identified in three districts and one county, from which five preschools were drawn randomly. All classes containing 543 children aged 5–6 years and their caregivers were included in the study. All children and their caregivers from the selected preschools received information about the goals and plans of our study. The study population consisted of children in three districts and one country of Changsha region (central China) and comprised 543 children aged 5–6 years and caregivers.

The level of ETS exposure of each child was estimated by analytical determination of cotinine levels in urine samples collected in the afternoon in the kindergarten. Each participant urinated into a polypropylene vial. A total of 50 mL urine was collected. Samples were frozen at −20 °C and packed in dry ice for shipping to the Hunan Academy of Inspection and Quarantine (Changsha, China). Analyses of cotinine concentrations were undertaken by gas chromatography–triple quadrupole mass spectrometry (GC-MS/MS) with a limit of quantification (LoQ) of 0.1 ng/mL. After 1 week, information about the smoking behaviors of caregivers at home as well as detailed information about the socio-demographic characteristics of children and their families was collected using face-to-face interviews. The average duration of the survey was 40 min.

### 2.3. Measurements

#### 2.3.1. ETS Exposure by Determination of Urinary Levels of Cotinine

Samples were coded and frozen at −20 °C until analyses. An analytical method for determination of urinary levels of cotinine in children exposed to ETS was established based on dilution of a stable isotope by GC-MS/MS. Samples were extracted and purified with chloroform. The internal standard (cotinine-d_3_) was added to urine samples and centrifuged at 7000 × g for 10 min at room temperature. Chromatography was conducted on a Restek Rxi-5 Sil MS column (30 m × 0.25 mm I.D., 25 μm; Shimadzu, Kyoto, Japan). The mass spectrometer was operated with a filament current of 150 μA and electron energy of 70 eV in electron impact ionization mode. Extracts were determined by GC–MS/MS in multiple reaction monitoring mode. Transition ions were monitored at *m/z* 176.10 > 98.05, *m/z* 176.10 > 147.05 and *m/z* 147.05 > 118.05 for cotinine, as well as *m/z* 179.10 > 101.10, *m/z* 179.10 > 147.05 and *m/z* 101.10 > 73.05 for cotinine-d_3_, respectively. Cotinine-d_3_ as an isotope internal standard was applied to quantify and confirm urinary levels of cotinine in children exposed to passive smoking. The method had good linearity from 0.1 ng/mL to 10 ng/mL with a correlation coefficient (*r*) of >0.998. The recovery of cotinine in blank urine was from 84.7% to 103.3% at spiked levels of 0.1, 1.0 and 10 ng/mL, respectively, with relative standard deviation from 2.1% to 5.8%.

#### 2.3.2. Self-Reporting of ETS Exposure of Children by Caregivers

The main measures that we looked at were: smoking in front of the child; smoking when the child was at home; smoking around the child at weekdays and at the weekend. The presence of caregiver smokers was assessed with the question, “Have you smoked a cigarette in this home (even a puff) since you moved into here?” The possible responses were “yes” or “no”. If the response was “yes”, the child was considered to be exposed to ETS, and the respondent invited to answer more questions: “How many smokers live in this home (including yourself)?” (an open numeric answer categorized in the analysis as 0 = no smoker; 1 = 1 smoker; 2 = more than 1 smoker) and “Does the smoker smoke near the child 3 m from the child?” (yes or no).

#### 2.3.3. Smoking Restrictions in the Home

Restriction of smoking at home could be “complete”, “partial” or “none”. Complete restriction of smoking was defined as no smoking at all within the home. Partial restriction of smoking was not smoking within 3 m of the child at home. No restriction of smoking was defined as unfettered smoking within the home.

Survey questions focused on smoking rules in the home. Caregivers answered the question on household rules about smoking (yes or no). If the response was “yes”, then the respondent was invited to answer other questions, such as “Are only certain people allowed to smoke within the home or is no one ever allowed to smoke inside the home, including yourself)” (yes or no).

#### 2.3.4. Self-Reporting of Smoking Status by Caregivers

We asked two main questions (which had open numeric answers): “On average, how many cigarettes are smoked by the smoker(s) during a weekday?” and “On average, how many cigarettes are smoked by the smoker(s) in the course of a weekday within the home?”

In our evaluation of how the intensity of the smoking behaviors of caregivers affected ETS exposure upon children, the combined total daily consumption of cigarettes was categorized: “light” consumption (1–10 cigarettes consumed daily); “moderate” consumption (11–20 cigarettes); and “heavy” consumption (>20 cigarettes).

### 2.4. Statistical Analyses

Statistical analyses were carried out using SPSS V20.0 (SPSS/IBM, Armonk, NY, USA). Non-parametric tests were used because the distribution of urinary cotinine values was non-Gaussian. Correlations between smoking status and urinary cotinine levels were tested using Spearman rank correlation tests. The Kruskal-Wallis test was used to explore differences in urinary cotinine levels. We compared values between children not living with a smoker and children living with one smoker; and children living with more than one smoker at home. The number of cigarettes consumed each day was not normally distributed. The Kruskal-Wallis test was used to examine differences in the mean number of cigarettes consumed in one day at home. The relationship between the number of smoker(s) in the household, daily cigarette consumption of the caregiver and urinary cotinine levels were tested using Nemenyi tests.

The stepwise linear regression analyses were conducted to estimate the independent effects of caregivers’ smoking behaviors on the urinary cotinine levels of children (urinary cotinine concentrations were naturally log-transformed). Variables entered into the model were the: caregiver’s age; size of the home; number of caregiver smokers (using children not living with the smoker as the reference group); smoking restriction (partial or no restriction); number of cigarettes smoked by the caregiver at home; smoker smokes at home every day and daily cigarette consumption. The variables were coded as follows:

“Daily cigarette consumption” was given as a categorical variable and coded in the regression analysis: (0–20 cigarettes/day = 0; >20 cigarettes/day = 1); domestic smoking restrictions (yes = 0; no = 1); caregiver smokes cigarettes at home each day (yes = 1; no = 0); and the number of caregiver smokers were analyzed as dummy variables (using children not living with the smoker as the reference group). Furthermore, the caregiver’s age and home size values were analyzed as continuous variables.

The two-tailed significance threshold chosen for all tests was *p* < 0.05. Linear regression analyses were conducted using a significance level of 0.05 for entry and a level of 0.10 for removal from models. The goodness of fit of the models was assessed using *R^2^*.

## 3. Results

### 3.1. Characteristics of Caregivers and Children

All of the 543 children and caregivers took part in the survey. All urine samples were collected and analyzed. Descriptive characteristics of the caregivers are given in Table 1.

**Table 1:** Characteristics of caregivers.

Characteristic	N	Value
Sex (%)		
male	510	93.92
female	33	6.08
Caregivers	543	
Mother	33	6.1
Father	477	87.8
Others (e.g., sibling, grandparent)	33	6.1
Age (years)	543	35.3 ± 6.6
Ethnicity (%)		
Han	541	99.63
other	2	0.37
Marital status (married *vs.* other) (%)	541	99.63
Income (<RMB 5999)	345	63.5
Home size (m^2^; mean ± SD)	543	111.4 ± 51.9
Number of cohabiting smokers (%)		
0	118	21.73
1	310	57.09
>1	115	21.73
Daily consumption of cigarettes (%)		
Light (1–10)	162	38.12
Moderate (11–20)	179	42.12
Heavy (>20)	84	19.76
Number of cigarettes smoked in one day by caregiver ^a^		
Overall	425	13.00 (6.00–18.00)
At home	425	6.00 (3.00–8.00)
Domestic smoking rules of caregiver (%)		
Total restriction	0	0.00
Partial restriction	11	2.03
No restriction	532	97.97

ETS, environmental tobacco smoke; RMB, Chinese renminbi. ^a^ Values are shown as the median and their interquartile range. Only includes caregiver smoking in front of children exposed to ETS.

The study cohort comprised 543 children, of which boys comprised 59.1% of participants. The sample of caregivers was unbalanced with respect to sex: >90% of caregiver respondents were males. The caregivers mainly included the father (87.8%, *n* = 477), mother (6.1%, *n* = 33) and others, such as grandparents or siblings (6.1%, *n* = 33). Most smokers in the Changsha families were fathers. Most caregivers spent time with their children both indoors (e.g., at home) and outdoors (e.g., car).

The mean age of children was 5.5 ± 0.6 years. The percentage of children who were exposed to ETS was higher than the percentage of children not exposed to ETS (78.3% *vs.* 21.7%). More than three-quarters of children lived with a smoker at home and approximately one-third of children had two caregivers in the household who smoked.

### 3.2. Children Exposed to ETS by Caregivers and Urinary Levels of Cotinine

**Figure 1:**
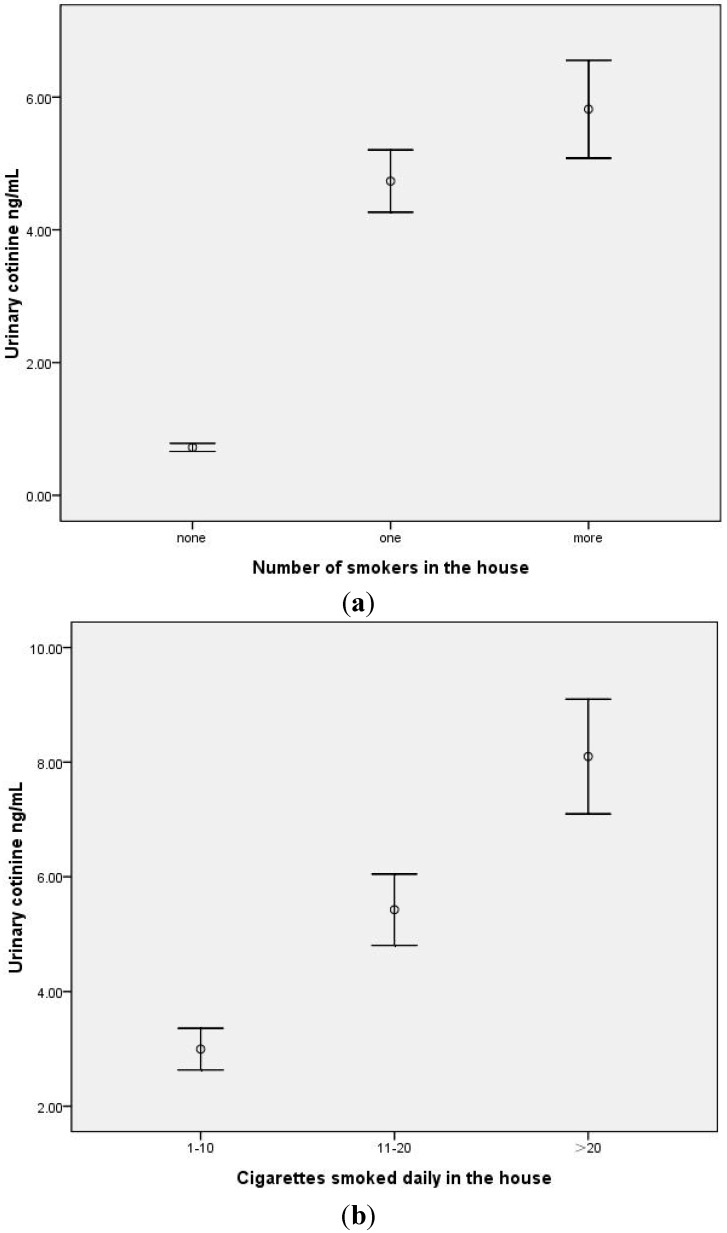
(**a**) Comparison between mean urinary cotinine (±SEM) according to the number of smokers within the house (none, 1, >1) (*p* < 0.001). There were significant differences between any two groups. (**b**) Comparison between mean urinary cotinine (±SEM) according to the mean number of cigarettes smoked every day within the house (<10, 11–20, >20 cigarettes (*p* < 0.001). There were significant differences between any two groups.

The association between urinary levels of cotinine for all children and ETS exposure groups is shown in Table 2. The median urinary cotinine concentration for the entire sample was 2.70 ng/mL (interquartile range = 1.76–4.76). The median urinary cotinine concentrations for children who did not live with a smoker, those living with one smoker, and those living with more than one smoker were 0.72, 2.97 and 4.46 ng/mL, respectively (*p* < 0.001) (Figure 1a). We examined the relationship between the number of smokers in the home and urinary cotinine levels of children (*r*_spearman_ = 0.665). The results suggested that there were significant differences between the groups of non-smokers and one smoker in the household (χ^2^ = 162.92, *p* < 0.001); there were also significant differences between the non-smoker and more than the one smoker groups in the household (χ^2^ = 229.03, *p* < 0.001), as well as the one smoker and more than one smoker groups in the household (χ^2^ = 15.29, *p* < 0.001).

**Table 2:** Association between urinary cotinine levels with characteristics of environmental tobacco smoke according to the smoking behaviors of caregivers.

Variable	N	Arithmetic Mean (ng/mL)	95% Confidence Interval	Median (ng/mL)	Interquartile Range	*p*	*r* *
No. of caregiving smokers	543						0.665 **
0	118	0.72	0.66–0.78	0.72	0.47, 0.91	<0.001	
1	310	4.73	4.27–5.20	2.97	2.12, 5.43		
>1	115	5.82	5.09–6.55	4.46	3.10, 7.31		
Smoking restriction	425						
Partial smoking restriction	11	4.25	1.92–6.58	2.55	2.21, 4.31	0.520	
No smoking restriction	414	5.05	4.65–5.45	3.43	2.29, 5.96		
Daily cigarette consumption of caregiver	425						0.573 **
light (1–10)	162	3.00	2.64–3.35	2.29	1.90, 2.71	<0.001	
moderate (11–20)	179	5.43	4.81–6.04	4.15	3.01, 5.85		
heavy (>20)	84	8.10	7.10–9.09	7.73	4.15, 10.67		

* Spearman correlation coefficient. ** p < 0.001.

All smoking households (73.3%) and non-smoking households (21.7%) reported no complete restrictions on smoking in the household. In contrast, 2.03% of smoking households had partial restrictions on smoking within the household. There was no difference in the urinary cotinine levels of children in whose house was a partial smoking restriction and of children in whose house was no smoking restriction in the household smoking (*p* = 0.520).

We examined the relationship between the mean number of cigarettes smoked in the home and urinary cotinine levels of children (*r*_spearman_ = 0.573, *p* < 0.001). The number of cigarettes smoked increased as the urinary cotinine levels of children increased (Table 2). With respect to the median values of urinary cotinine levels of children, there were differences between the three groups of smokers: light = 2.29 ng/mL; moderate = 4.15 ng/mL; and heavy = 7.73 ng/mL, respectively. There were differences in the urinary cotinine levels of children in whose house were smokers with different levels of daily consumption of cigarettes (Figure 1b). There were significant differences between the light and moderate exposure groups (χ^2^ = 114.28, *p* < 0.001), and the light and heavy groups (χ^2^ = 93.71, *p* < 0.001) as well as the moderate and heavy groups (χ^2^ = 21.38, *p* < 0.001).

The stepwise linear regression model (Table 3) showed that children living with one smoker (B = 1.256; *p* = 0.000) or living with more than one smoker (B = 1.481; *p* = 0.000) showed significant differences to children that did not live with a smoker. Additionally, the daily cigarette consumption of caregivers (B = 0.647; *p* = 0.000) and caregivers smoking cigarettes at home each day (B = 0.330; *p* = 0.008) were important predictors of urinary levels of cotinine in children, adjusting for the variables of caregivers’ age and home size. Thus, the models accounted for variability in urinary cotinine levels of 64.8%.

**Table 3:** Differences in urinary cotinine levels for children living with smokers and the smoking behaviors of the caregiver.

Independent Variable	Beta (Unstandardized Coefficient)	Std Error *	Beta (Standardized Coefficient)	*R*^2^	*t*	*p*
MODEL ^a^				0.650		
Children not living with smoker (reference group )	1					
Children living with one smoker	1.256	0.134	0.650		9.403	0.000
Children living with more than one smoker	1.481	0.139	0.633		10.640	0.000
Daily cigarette consumption (>20)	0.647	0.070	0.245		9.302	0.000
Smoker smokes at home every day	0.330	0.125	0.151		2.650	0.008

^a^ Final stepwise linear regression model with the caregiver’s age, home size, number of caregiver smokers, domestic smoking restrictions, if the caregiver smokes cigarettes at home every day, and daily cigarette consumption of caregiver; constant = −0.388; final adjusted *r* = 0.648. * Std error: standard error.

## 4. Discussion

### 4.1. A High Proportion of Preschool Children Are Exposed to ETS at Home

Our findings have shown that approximately four-fifths of children live with at least one smoker at home, a prevalence that is higher than that shown in other countries [23,24,25]. This prevalence is not consistent with that found in other studies, such as 43.6% in US and 27.4% in UK [26,27]. The impact of the number of smokers at home on ETS exposure to children was highlighted by the significant and progressive increases in urinary cotinine levels in children not living with a smoker to children, living with one smoker, and those living with more than smoker, in the house. This finding is in agreement with other studies [28,29]. These are findings based on large and representative samples, as well as the availability of cotinine values to validate domestic smoking restrictions and quantify the impact of ETS exposure upon children.

We found that the urinary cotinine levels of children living with a smoker increased in direct proportion to the intensity of smoking behaviors of the caregiver. This finding was very important for children who live with a caregiver who smokes a lot of cigarettes every day at home. Consuming >20 cigarettes/day was found to be significant predictive factor of higher urinary cotinine levels in children. However, there is no risk-free level of exposure in environmental tobacco smoke, more smoking by caregivers lead to higher urinary cotinine levels of children. The very high prevalence of smoking it is very hard to protect children. Self-reported measures of exposure showed moderate correlation with urinary levels of cotinine of children, and could explain the variability in cotinine levels. It is plausible that a caregiver with a high nicotine dependency would find it extremely difficult not to smoke anywhere, and is therefore less likely to be able to restrict his/her household smoking restrictions. Another study found that smokers who consumed ≥11 cigarettes/day were less likely to restrict smoking at home [30]. This relationship showed that heavy smokers, in addition to risk-adverse effects on their health, are endangering children and non-smoking adults in the same environment.

Urinary cotinine concentrations are commonly corrected for creatinine concentrations to adjust for differences in urine flow [31]. However, a study using liquid chromatography coupled with tandem mass spectrometry to measure urinary cotinine concentrations in children passively exposed to ETS found that creatinine-corrected urine cotinine concentrations correlated less well with parental smoking history than did uncorrected values [32]. Moreover, some studies have suggested that dietary exposure to nicotinine may confound the low-level determination of nicotinine and cotinine in biologic fluids. Nicotinine is present in some foods but, at usual food-consumption levels, exposure to dietary nicotinine is trivial compared with moderate exposure to ETS [33]. These observations are in accordance with the urinary cotinine levels we found in the group of children not living with a smoker.

### 4.2. Smoking Restrictions in Families Were Low

A partial domestic smoking ban was reported by 2.03% of our Changsha caregivers, which is lower than that noted in other studies [34,35,36,37]. Several factors may have contributed to this disparity. First, the prevalence of cigarette smoking is much higher among men (60.2%) than among women (6.9%) in 2004 [38]. Therefore, most of the participants in our study cohort were male caregivers. Second, smoking (especially among men) is accepted widely in China. However, it is well-known that most Chinese women, especially mothers, do not smoke [39]. Third, Chinese people have a tradition of providing cigarettes, drink and tea to guests as a sign of hospitality. Fourth, by enforcing certain smoking restrictions, caregivers may have felt that they were being hypocritical, especially if they could not provide cigarettes for guests. Fifth, there appeared to be a conflict between being a smoker and being a caregiver. The dominant role of males in Chinese society can aid understanding why most households failed to apply any domestic smoking restriction (especially if the head of the household is a male smoker). Overall, it is not surprising that many families did not adopt any smoking restriction. However, ban smoking in public places was a major step forwards in reducing ETS exposure outside the home.

The private domain is the important locations of exposure to ETS for children. However, reducing exposure in this domain is difficult to achieve [40]. Currently, strategies to reduce children’s exposure to tobacco smoke fall into two categories: (i) helping caregivers to stop smoking and (ii) keeping children away from ETS. One of the most effective ways of lowering the impact of ETS on children is smoking cessation by parent(s), especially by the father, in China. It is difficult to stop smoking, but changing location and increasing knowledge of ETS are practicable steps by smoking caregivers to changing smoking behavior and, ultimately, stopping smoking. Such steps would suggest contemplation or preparation to stop smoking. Future interventions should consider special methods to ensure involvement from smokers, including self-help materials, face-to-face counseling, and tobacco dependence treatment medications.

### 4.3. Strengths and Limitations

The present study is first to examine the impact of domestic smoking behaviors of caregiver smokers on a large sample of Chinese children aged 5–6 years using a proven biomarker of exposure to ETS (cotinine) as an objective parameter. Nevertheless, our study had limitations. Firstly, we did not test the urine cotinine to creatinine concentration ratios and unmeasured variation in urine concentration could decrease precision of estimates it; moreover, urinary cotinine concentrations may be affected by the level of ventilation and the daily activities of children, for which we did not collect data. Secondly, the data suggested that domestic smoking restrictions were minimal at home. The results of our study show that ETS exposure in families in Changsha was very common. It may be argued by some scholars that developing interventions and strategies to protect children from ETS exposure would be difficult in families. However, our finding points to the need to develop strategies that can help caregivers to reduce children’s exposure to ETS, with consideration that socio-cultural factors might influence their effectiveness. Third, urine samples were collected only once from each child, so possible changes in urinary cotinine levels over time could not be observed. Studies have indicated that cotinine detection from single samples of urine provide highly accurate estimates of a child’s exposure in the past 2–3 days [21]. Fourthly, commentators have pointed to the newly defined issue of “third-hand” smoke, which is the residue from tobacco smoke that persists on the clothing and hair of smokers, on environmental surfaces, and in dust long after a cigarette has been extinguished [3,14,41]. In future studies, ETS exposure as measured by airborne PM_2.5_ will be added as supplemental data.

## 5. Conclusions

Our findings suggest that the urinary cotinine levels of Chinese children exposed to environmental tobacco smoke are positively correlated with the daily smoking behaviors of their caregivers in the household. A high proportion of smoking caregivers with 5–6-year-old children displayed poor domestic smoking restrictions. Counseling for smoking cessation and educational interventions are needed urgently for such smoking caregivers.
